# Single-cell analysis of matched FFPE and frozen tissue samples reveals comparable resolution of intratumoural heterogeneity

**DOI:** 10.3389/fgene.2026.1755978

**Published:** 2026-03-24

**Authors:** Cathy Yan, Richard D. Corbett, Diane Trinh, Dan Jin, Janessa Laskin, Melanie L. Bailey, Marco A. Marra

**Affiliations:** 1 Genome Science and Technology Graduate Program, University of British Columbia, Vancouver, BC, Canada; 2 Michael Smith Laboratories, University of British Columbia, Vancouver, BC, Canada; 3 Canada’s Michael Smith Genome Sciences Centre, Provincial Health Services Authority, Vancouver, BC, Canada; 4 Department of Medical Oncology, BC Cancer, Vancouver, BC, Canada; 5 Department of Medical Genetics, University of British Columbia, Vancouver, BC, Canada; 6 BC Cancer Research Institute, BC Cancer, Vancouver, BC, Canada

**Keywords:** cancer, formalin-fixed paraffin-embedded tissue(FFPE), intra-tumoural heterogeneity, single-cell RNA sequencing (scRNA-seq), tumour microenvironment

## Abstract

**Introduction:**

Intra-tumoural heterogeneity contributes to treatment resistance and disease progression in cancer. Single-cell RNA sequencing (scRNA-seq) enables profiling of cellular diversity in the tumour microenvironment. However, current protocols for generating single-cell 3′gene expression data require intact RNA, which excludes archival formalin-fixed paraffin-embedded (FFPE) tissues. A commercial method now enables scRNA-seq on FFPE samples using a probe-based approach. While methods for spatial and single-cell profiling on FFPE have been previously assessed, none have evaluated whether intra-tumoural heterogeneity was comparable between scRNA-seq data from matched FFPE and fresh-frozen cancer samples across a range of cancers.

**Methods:**

scRNA-seq was performed on 12 pairs of matched FFPE and frozen tumour tissue and results were compared across key stages of a standardized analysis pipeline. This included cell type annotation, identification of malignant cells, optimization of batch correction methods, and characterization of immune cell subtypes.

**Results:**

ScRNA-seq from fresh frozen material yielded higher median numbers of unique molecular identifiers (UMIs) and genes per cell than data from FFPE tissues. However, across both FFPE and frozen samples, the same cell types were consistently identified, and candidate malignant cells could be detected by inferring copy number alterations (CNAs). FFPE-derived data were able to resolve sub-clonal CNAs and characterize T cell subpopulations.

**Discussion:**

These findings demonstrate that intra-tumoural heterogeneity can be inferred from FFPE-based scRNA-seq using tissue samples typically prepared in hospital pathology laboratories. By enabling analysis of widely available FFPE specimens, this approach opens new avenues for population-scale single-cell profiling in cancer research.

## 1 Introduction

Intra-tumoural heterogeneity (ITH) is a fundamental property of malignancies that drives tumour evolution, therapeutic resistance, and disease progression in cancer (Marusyk et al., 2012). The presence of genetically or phenotypically distinct subpopulations of cancer cells within tumours presents significant theoretical and practical obstacles to curative treatment, as therapies may eliminate sensitive subclones while allowing resistant subclones to survive (Fu et al., 2025). In addition to malignant cells, the presence of immune cells in the tumour microenvironment (TME) has been shown to predict therapeutic efficacy (Liu Y. et al., 2024), with the abundance and phenotypes of T cells implicated in immunotherapy efficacy (Zhou et al., 2022). Therefore, resolving ITH and the TME is a key consideration in the development of effective therapeutic strategies.

Single-cell sequencing is a powerful approach for dissecting tumour cellular ecosystems. However, it often requires intact cellular or nuclear RNA from fresh or fresh-frozen tissue. Unlike FFPE tissues, fresh or fresh frozen tissues can be difficult to obtain in routine clinical settings due to logistical constraints, such as the need to rapidly process biopsies for clinical testing (Cho et al., 2017; Wiegleb et al., 2022). Consequently, single-cell profiling of fresh or fresh frozen tissue samples from hospital pathology laboratories is generally not possible. In contrast, FFPE samples are routinely collected and archived in clinical practice, offering a substantial repository of diverse tumour specimens and a potentially abundant resource for cancer genomics (Mathieson and Thomas, 2020).

Although the fixation process introduces molecular biology challenges such as nucleic acid degradation and chemical modifications, genomic and transcriptomic profiling from FFPE material is achievable (Robbe et al., 2018; Haile et al., 2019). In addition to bulk methods (Turnbull et al., 2020), protocols have also been developed to profile the transcriptome and epigenome of FFPE tissues at single-cell and/or spatial levels (He et al., 2022; Xu et al., 2023; Wang et al., 2024), an example of which is the 10X Genomics Chromium Fixed RNA Profiling (Flex) platform (Flex Gene Expression, 2025). In the context of large-scale precision oncology programs, such as the Personalized OncoGenomics (POG) program at Canada’s Michael Smith Genome Sciences Centre (Pleasance et al., 2020; Pleasance et al., 2022), a commercially-available, high-throughput method capable of profiling archival tumour samples, such as Flex, may improve the quality and utility of the RNA profiling data that can be obtained. However, it is not yet known to what extent ITH can be captured using the Flex platform for diverse cancer samples from different anatomical sites. Here we compared single-cell data generated from matched pairs of FFPE and frozen samples from 12 different cancer samples from seven POG patients. We ultimately find that data from FFPE Flex is comparable to, and can be integrated with, data from 3′ scRNA-seq, demonstrating the potential of analyzing single-cell profiles from FFPE samples, such as those typically prepared in a clinical setting.

## 2 Methods

### 2.1 Ethical oversight, consent, and enrolment

The POG program is a single-arm prospective trial (clinical trial number NCT02155621) approved by the University of British Columbia - BC Cancer Research Ethics Board (H12-00137 (July 2012 to July 2014), H14-00681 (July 2014 to July 2020), and H20-02317 (July 2020 to present)). Patients were enrolled by medical oncologists from across British Columbia, Canada, and provided written informed consent.

### 2.2 Sample selection

All participants were diagnosed with advanced or metastatic cancer and had previously undergone bulk WGS and WTS as described in Pleasance et al. (2022). Samples for this retrospective analysis were selected based on availability of matching fresh-frozen/OCT-embedded tissue suitable and FFPE blocks from the same biopsy and surgical site. Tissue that had been previously embedded in OCT for pathology and diagnostic analysis were not excluded from our cohort although excess OCT had to be removed before being used for scRNA-seq. Additionally, samples had to meet one or more of the following criteria: 1) multiple biopsies were available from the same patient, 2) tumour content was predicted to be below 50%, and/or 3) there was suspected high immune infiltration based on deconvolution of bulk WTS.

### 2.3 Single-cell library construction

Twelve fresh-frozen and matching FFPE samples from the same biopsy were obtained from the POG program at Canada’s Michael Smith Genome Sciences Centre (Supplementary Table S1). All samples were de-identified and assigned a POG ID (POGXXXX) before this study was undertaken. Patients with multiple biopsies are labelled with a single POG ID but different biopsy numbers, where the lowest biopsy number representing the earliest sample. Excess OCT was removed from fresh frozen/OCT-embedded tissue. Approximately 25–100 mg of tissue was processed using the Chromium Nuclei Isolation Kit with RNase Inhibitor (10X Genomics) to prepare nuclei according to the Multiome specifications in the Sample Prep User Guide (CG000505). Nuclei were counted using DAPI and an EVOS M5000 microscope on disposable counting slides. Up to 10,000 nuclei were targeted for single cell library construction using the Chromium Next GEM Single Cell Multiome ATAC + Gene Expression kit (10X Genomics) according to the manufacturer’s recommendations (User Guide CG000338).

For FFPE tissue, 4-9 sections of 20 µm thickness were dewaxed and homogenized for single nuclei isolation according to the 10X Genomics Demonstrated Protocol (CG000632). Nuclei were counted as above and ∼140,000–900,000 nuclei were used for probe hybridization with the Chromium Fixed RNA Profiling Reagent Kit (10X Genomics). Subsequent GEM generation (targeting 10,000 nuclei) and library construction were performed according to the manufacturer’s instructions (CG000691). All constructed libraries were subjected to both Agilent Bioanalyzer profiling and Qubit quantification before being sequenced using PET150bp sequencing on an Illumina NovaSeqX at Canada’s Michael Smith Genome Sciences Centre (Vancouver, Canada). At least 20,000 read pairs/nucleus were sequenced for the 3′scRNA-seq libraries from the Multiome kit and at least 10,000 read pairs/nucleus were sequenced for the Fixed RNA/Flex libraries.

### 2.4 Computational analyses

#### 2.4.1 Quality control

Sequencing results from these experiments yielded wide ranges of read pairs per nucleus (FFPE: 9136-120,495; Fresh/OCT: 19,713-83,330) and raw sequence yield per sample (FFPE: 60 × 10^6^–150 × 10^6^ Fresh/OCT: 160 × 10^6^–280 × 10 (Wiegleb et al., 2022). With no significant Pearson correlation in read count between matching samples ( ρ = −0.21, p-value = 0.43), each pair of fastq files were down-sampled to 60 million to control for depth variability. The resulting FASTQ files from both the FFPE and fresh frozen tissue samples were analyzed using CellRanger-9.0.0 with the GRCh38 ensembl 98 reference. The outputs were loaded into R (v4.2.2) for analysis using Seurat (v5.2.1). Cells which met one or more of the following criteria were excluded from the final dataset (Amezquita et al., 2022): 1) cells with a UMI count greater than three mean absolute deviations below the sample median, 2) cells with a gene count greater than three mean absolute deviations below the sample median, 3) cells where more than ten percent of UMIs mapped to mitochondrial genes, and/or 4) those identified as doublets by scDblFindr (v1.12.0) (Germain et al., 2022).

#### 2.4.2 Cell type annotation

Cell type annotation was performed by mapping labels from Guimarães et al. (2024), a pan-cancer scRNA-seq atlas, using Seurat’s FindTransferAnchors and TransferData functions. A Seurat object containing raw counts and metadata for the reference was downloaded from CZI CELLxGENE (Megill et al., 2021). These data were processed as described in Guimarães et al. (2024). To validate trends in prediction confidence scores, cell type annotation was also performed using SingleR (v2.0.0) and the Human Primary Cell Atlas (Mabbott et al., 2013). Each sample in our study was annotated independently. T cells were further subtyped and assessed for markers of exhaustion (PDCD1, CTLA4, HAVCR2, LAG3, TIGIT, and TOX) (Jenkins et al., 2023). T cells were clustered and annotated using a combination of canonical marker genes (Mullan et al., 2023) and through assessment of differentially expressed genes for each cluster.

To identify candidate malignant cells and malignant cell clusters, inferCNV (v1.3.3) was used to infer copy number alterations (CNAs). Cells annotated as immune cells were used as the reference population. For each cell, CNA predictions were taken at the gene level and averaged across windows of 10 Mb. The same was done to predict CNAs from bulk whole genome sequencing (WGS) data, which were identified as described in (Pleasance et al., 2022). To compare single-cell and WGS data, CNAs were classified as “gain” (score >1), “neutral” (1), or “loss” (<1). For each cell, Cohen’s kappa was used to quantify the degree to which single-cell and bulk CNA profiles were similar. Single cell clusters where the 10th percentile of the absolute value of CNA scores was greater than 0 were classified as malignant. To assess whether a population of cells has a gain or loss, a one-sample sign test was performed for the respective direction, with 1 as the population mean.

#### 2.4.3 Batch correction and clustering

Batch correction and clustering parameters were optimized for each FFPE-fresh frozen tissue pair by iterating over combinations of the batch correction methods available in Seurat (Hao et al., 2024). These methods included Harmony (Korsunsky et al., 2019), FastMNN (Haghverdi et al., 2018), canonical correlation analysis (CCA), joint principal component analysis (JPCA), and reciprocal principal component analysis (RPCA). For clustering, the resolution ranged from 0.05 to 0.3 in increments of 0.05. The higher the resolution, the greater the number of clusters. Clustering parameters were selected using normalized mutual information (NMI) (McDaid et al., 2013), as described in Tran et al. (2020).

FFPE Flex and 3′ scRNA-seq data were randomly sampled 150 times to generate subsets of varying overlap. Twenty-five of these yielded “complete overlaps” where all cell types in both FFPE Flex and 3′ scRNA-seq were the same. An additional twenty-five had no overlaps in cell types between the two platforms. In one hundred scenarios, the two modalities had some shared cell types (partial overlap). These datasets were generated by first randomly selecting samples and cell types for partitioning the 3′ scRNA-seq. For complete overlap, the exact same samples and cell types were used to subset the FFPE Flex. For partial overlap, another random selection of samples and cell types was performed. For no overlap, only cells belonging to samples and cell types not selected were extracted from the FFPE Flex dataset.

We assessed batch correction using four metrics used to evaluate the integration of single-cell atlases (Tran et al., 2020). NMI and adjusted Rand Index (ARI) (Hubert and Arabie, 1985) were used to assess congruence between cluster identities and cell types. Adjusted silhouette width (ASW) (Rousseeuw, 1987) and principle component regression were used to evaluate mixing across platforms. All methods were implemented as described in Tran et al. (2020). For NMI and ARI, a higher value denotes higher correspondence between clusters and cell types, implying stronger conservation of cell type biology. For ASW and principal component regression, lower scores indicate better mixing across platforms. As ASW requires cells from both platforms to be present in each cell type, it cannot be computed for the “no overlap” category.

### 2.5 Statistics

Statistical comparisons were performed using glmmTMB (v1.1.11). For cell type prediction scores and Cohen’s kappa, linear models were fitted accounting for the sample from which the cells came. Resulting p-values were corrected using Benjamini–Hochberg false discovery rate correction (FDR). To compare cell type composition between fresh frozen and FFPE samples, the Cochran-Mantel-Haenszel test was used.

## 3 Results

### 3.1 Cohort overview

**FIGURE 1:**
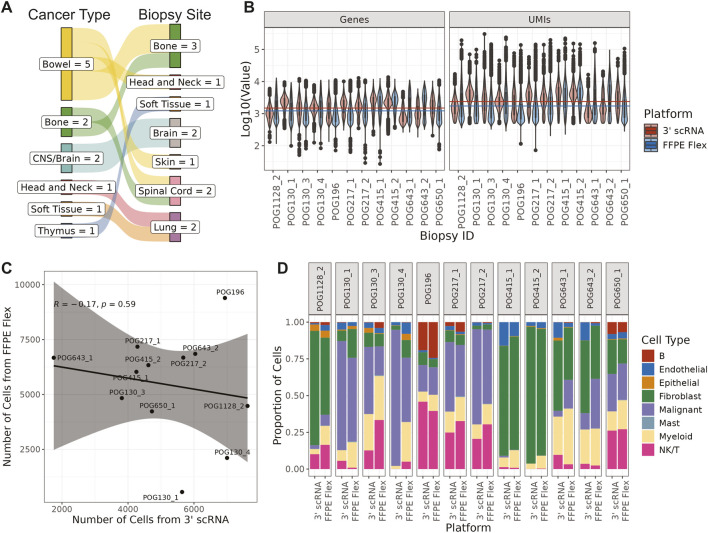
Sample overview **(A)** Sankey plot showing the diversity of cancer types in the cohort, and the sites from where tumours were biopsied **(B)** Violin plots comparing the distributions of the number of genes (left) and the number of UMIs (right) detected in each cell. Horizontal lines denote the median values across the cohort for each single-cell profiling platform **(C)** A comparison between the number of cells identified from 3′ scRNA and FFPE Flex data after quality control filtering (Pearson’s correlation coefficient = −0.17, p-value = 0.59) **(D)** Stacked bar plot showing the proportion of each cell type found in each sample (Cochran-Mantel-Haenszel test p-value <0.001).

Twelve metastatic, solid tumour samples from seven patients were included in this study (Supplementary Table 1; Figure 1A). Each patient provided matched fresh frozen (with or without optimal cutting temperature (OCT) compound embedding), and FFPE tissue from the same biopsy. Samples ranged in age from 2.5 to 10 years old. On average, there were 5,446 cells (IQR: 4,416–6,721 cells) called by Cell Ranger from FFPE libraries, and 5,197 cells (IQR: 4,278–6,264 cells) from 3′ scRNA seq. Generally, FFPE samples had fewer genes and UMIs per cell (Figure 1B), potentially because the Flex platform has probes for fewer genes than the genes included in the mapping reference for 3′ scRNA-seq data (Methods, Supplementary Table 2). After quality control, there was no significant relationship between the number of cells from fresh frozen tissue and the corresponding number from FFPE tissue (Pearson’s correlation coefficient = −0.17, p-value = 0.59; Figure 1C; Supplementary Table 3). However, we did find that the proportion of cell types differed significantly across matched pairs (Cochran-Mantel-Haenszel test p-value <0.001; Figure 1D).

### 3.2 Cell types are called with higher confidence from FFPE flex

**FIGURE 2:**
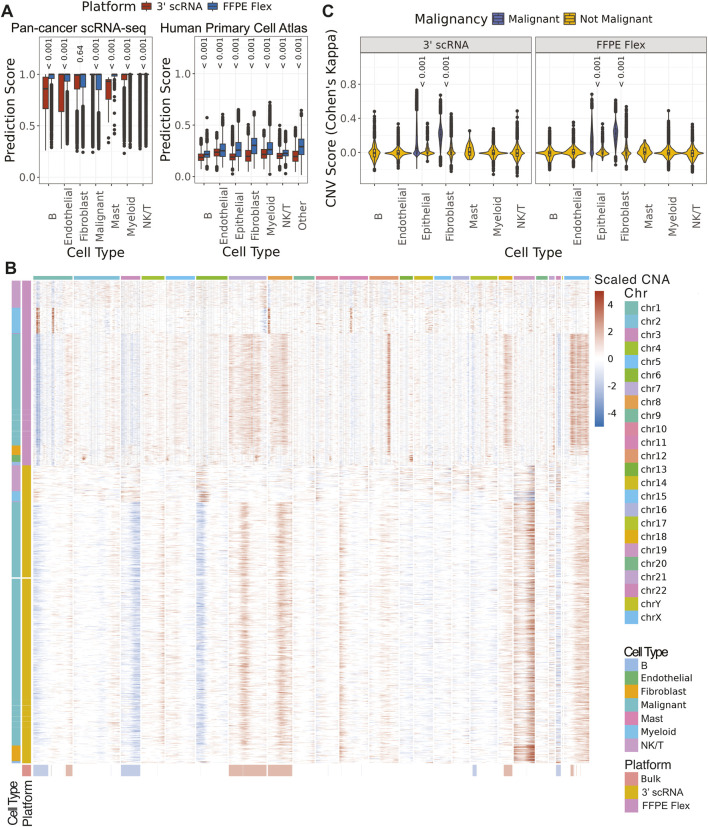
Cell types were assigned with greater confidence using FFPE Flex data **(A)** The distribution of prediction confidence scores by cell type across all cells in the cohort using two different methods and references (Guimarães et al., 2024; Mabbott et al., 2013) **(B)** CNA scores for malignant and non-malignant cells were compared across all cells in the cohort **(C)** Heatmap of predicted CNA values from 3′ scRNA-seq data, FFPE Flex data, and bulk WGS for POG1128 as an example.

After combining cells from all samples, we found that cell types called using FFPE data were assigned with higher confidence scores compared to cell types called using fresh frozen tissue (FDR <0.001), except for malignant cells and fibroblasts when using Seurat with Guimarães et al. (2024) as a reference (Figure 2A). Cell types in our FFPE data were assigned higher confidence scores when using SingleR with the HPCA reference (FDR <0.001). To differentiate between malignant and normal fibroblasts in the three sarcoma samples (Supplementary Table 1), and to validate malignant epithelial cell calling, somatic CNAs were inferred (Methods). All samples also had bulk whole genome sequencing (WGS) and transcriptome sequencing data available from the Personalized OncoGenomics (POG) program at BC Cancer (Pleasance et al., 2020; Pleasance et al., 2022). Somatic CNAs from bulk WGS were used as the reference to which inferred fresh frozen CNAs were compared (Figure 2B; Supplementary Figure 1). Analyses using Cohen’s Kappa revealed that malignant epithelial and fibroblast populations had significantly higher scores than normal cells (FDR <0.001) in both fresh and FFPE single cell data (Methods, Figure 2C), indicating that inferred CNAs are viable for detecting malignancy.

### 3.3 Integrating FFPE flex and 3′ scRNA-seq data

**FIGURE 3:**
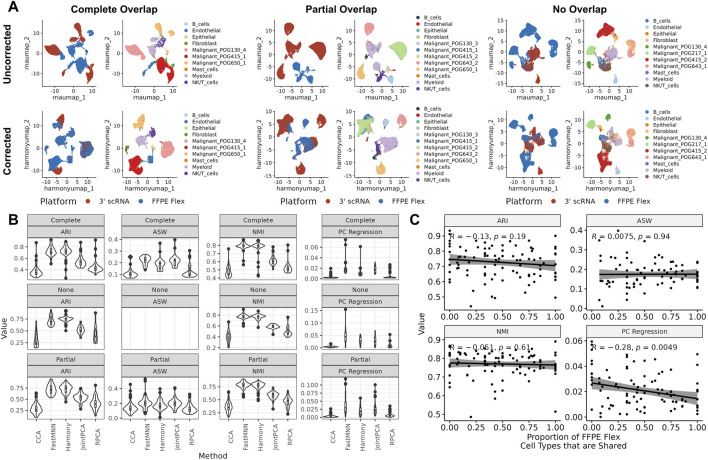
FFPE Flex data can be integrated with 3′ scRNA **(A)** UMAPs of three example scenarios with varying levels of shared cell types coloured by platform (left) and cell type (right) before and after batch correction with Harmony **(B)** Box and violin plots comparing scores across different scenarios and batch correction methods **(C)** Scatterplots with Pearson’s correlation coefficient and p-values depicting relationships between the amount of cell type overlap and metrics for Harmony.

To determine the extent to which FFPE single cell data could be integrated with fresh-frozen tissue data, the cohort was randomly partitioned to generate FFPE Flex and 3′ scRNA-seq datasets with varying degrees of overlap in cell type composition (Methods, Figure 3A). These datasets were iteratively processed across different batch correction parameters (Methods). Outputs were assessed for conservation of cell types using NMI and ARI, and integration across platforms using ASW and principal component regression. FastMNN and Harmony consistently resulted in higher ARI and NMI scores across all scenarios, indicating stronger bio-conservation (Figure 3B). FastMNN did not mix cells across platforms as well as other methods, with higher ASW and PC regression values. Thus, we found that Harmony was the best method FFPE Flex and 3′ scRNA-seq integration. Additionally, we sought to examine if batch correction effectiveness was impacted by the extent to which cell types were shared across platforms. Only PC regression scores had a low, negative correlation with the proportion of shared cell types (Figure 3C; Pearson’s R = −0.28, p-value = 0.0049). This suggests that Harmony works well regardless of cell type similarity between FFPE Flex and 3′ scRNA-seq datasets.

### 3.4 FFPE flex resolves T cell subtypes and malignant subclones

#### 3.4.1 T cell subtyping and quantification

**FIGURE 4:**
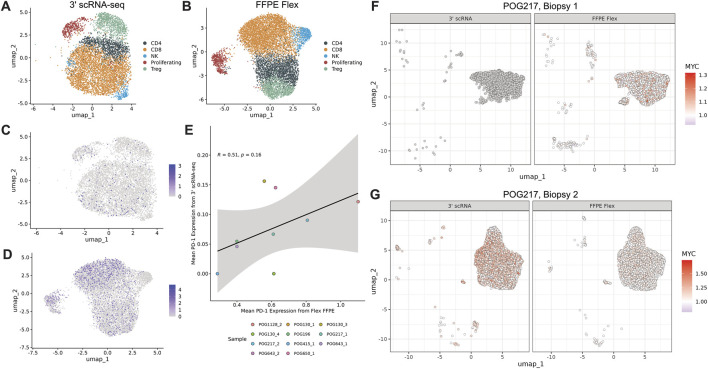
FFPE Flex can detect clinically relevant biomarker expression **(A, B)** Batch corrected UMAPs of T cells from all samples coloured by T cell subtype from **(A)** 3′ scRNA and **(B)** FFPE Flex **(C, D)** UMAPs of T cells coloured by PDCD1 expression from **(C)** 3′ scRNA and **(D)** FFPE Flex **(E)** Scatterplot showing cross-platform correlation of average PDCD1 expression across samples **(F, G)** UMAP of malignant cells coloured by inferred MYC CNA status from **(F)** 3′ scRNA and FFPE Flex in biopsy 1 (dark grey dots represent cells for which MYC CNA status could not be inferred) and **(G)** biopsy 2.

We assessed the extent to which FFPE data could infer T cell subtypes, which are a frequently studied component of ITH. After T cells from each platform were clustered and annotated based on expression of known marker genes (Supplementary Figures 2A–D; 12), T cell populations were labelled (Figures 4A,B) in both FFPE and fresh frozen data. To affirm that a clinically relevant aspect of CD8^+^ T cell biology was comparable, we found that PDCD1 expression was detectable in both fresh-frozen and FFPE datasets, with the latter having more cells expressing the gene at higher values (Figures 4C,D). However, PDCD1 expression across CD8^+^ T cells in each sample did not correlate significantly (R = 0.51, Pearson’s p-value = 0.16; Figure 4E).

#### 3.4.2 Tracking subpopulations across biopsies

Beyond the presence of different cell types, we also sought to examine heterogeneity across the malignant cell population. Within this cohort, there were three sets of longitudinal biopsies from the same patient. Based on bulk WGS, one set, POG217, had a MYC amplification that was only present in the second biopsy. When investigating whether this amplification was detectable subclonally in the first biopsy, we found that the fresh frozen data lacked resolution at our arbitrarily selected down sampled sequencing depth for inferring MYC amplifications (Figure 4F). The FFPE data, however, were able to infer subclonal amplification of MYC in the first biopsy (one-sample sign test p-value <0.001). Both the fresh frozen (one-sample sign test p-value <0.001) and the FFPE (one-sample sign test p-value <0.001) datasets were able to infer this amplification in the second biopsy due to its increased prevalence (Figure 4G).

## 4 Discussion

In this study, we demonstrated that single cell expression analysis using FFPE samples can infer cell type composition and characteristics that are comparable to single cell RNA seq analysis method using fresh frozen tissues. Although other studies have performed similar comparisons using their own protocols (Wang et al., 2024; De Simone et al., 2024), ours is the first to use commercially available kits to examine archival patient samples from seven different anatomical sites (Figure 1A; Supplementary Table 1). Overall, we found that, despite differences in sample preparation and overall data quality, FFPE-derived data preserved features of tumour biology seen in fresh-frozen samples, including cell type identification and inference of malignant states via CNAs.

The 3′scRNA-seq data derived from fresh frozen and OCT samples generally outperformed FFPE-derived Flex data in metrics such as the number of UMIs and genes detected per cell (Figure 1B), which was expected as Flex probes query half as many genes as 3′ scRNA-seq generally detects (Supplementary Table 2). Regardless, confidence scores for cell type annotation were often higher in FFPE samples using two different annotation methods and references (Figure 2A). This may reflect the targeted nature of the Flex probe set, which might reduce noise that could obscure cell type marker genes. Furthermore, as all datasets were down-sampled to the same total number of read pairs (Methods), the number of reads per gene was higher for FFPE Flex than 3′ scRNA-seq. This may also have improved cell type annotation. For sarcoma samples, CNA inference was used to distinguish between normal and malignant fibroblasts (Figure 2C). There was strong concordance between scRNA-seq-inferred CNAs and matched bulk whole-genome sequencing data, even for sparse CNA profiles inferred from the Flex data.

Despite these strengths, the integration of FFPE and fresh frozen datasets required optimization. After partitioning our cohort to mimic possible scenarios for batch correction (Figure 3A), we systematically evaluated methods based on metrics for bio-conservation and cross-platform mixing. We found that Harmony consistently performed the best (Figure 3B), regardless of how many cell types were shared across datasets (Figure 3C). After batch correction, we demonstrated that immune system features remained discernible in FFPE-derived single-cell data. For example, we inferred T cell subtypes (Supplementary Figure 2A–D, Figure 4A, B), and observed heterogeneous expression of PDCD1 (Figure 4C–E), an important marker for immunotherapy efficacy (Liu R. et al., 2024). Additionally, across multiple biopsies taken longitudinally from the same patient, sub-clonal amplification of MYC was detectable using FFPE Flex, even though sequencing coverage was insufficient for predicting MYC’s CNA status in one of the 3′ scRNA-seq samples. In summary, our approach to integration serves as a framework for merging data from diverse preservation modalities.

This study has several limitations. First, although FFPE and fresh frozen or OCT pairs came from the same biopsy, they are from different segments of tissue and thus contain different cells, leading to differences in the cell populations we profiled. Additionally, different preservation methods may introduce biases in gene expression (Denisenko et al., 2020), which we do not explicitly address in this manuscript. The probe-based nature of Flex also constrains transcriptome coverage to predefined regions of transcripts that are fewer in number than what is detectable through 3′ scRNA-seq (Supplementary Table 2). Finally, although batch correction strategies were evaluated in this study, we were not able to test the scalability of these methods to larger, more heterogeneous cohorts. Comparisons to tools used in clinical workflows such as Affymetrix microarrays, and other single-cell platforms including Illumina’s are also potential avenues of future study.

Our findings demonstrate that scRNA-seq on FFPE tissues using the 10X Genomics Flex platform is capable of inferring ITH, including cell identity and sub-clonal genomic alterations, comparable to 3′ scRNA-seq on fresh frozen tissues. The broad accessibility of FFPE tissues, including from archived samples, position this technology as a powerful tool to unlock single-cell insights across diverse cancer types, patient populations, and treatment histories, advancing the integration of high-resolution tumour biology into precision oncology.

## Data Availability

The original contributions presented in the study are publicly available. This data can be found in the European Genome-phenome Archive (EGA) repository with the accession number EGAD00001015781, at https://ega-archive.org/datasets/EGAD00001015781.
