# Supplementary material for: Single-cell analysis of matched FFPE and frozen tissue samples reveals comparable resolution of intratumoural heterogeneity
Source: Front Genet. 2026 Mar 24;17:1755978. doi: 10.3389/fgene.2026.1755978 (PMC13055531; doi:10.3389/fgene.2026.1755978)
Supplement: Supplementary file 1 [file Supplementaryfile1.docx]

Supplementary Material

Single-cell analysis of matched FFPE and frozen tissue samples reveals comparable resolution of intratumoural heterogeneity

Cathy Yan^1,2,3^, Richard D. Corbett^2,3^, Diane Trinh^2,3^, Dan Jin^2,3^, Janessa Laskin^4^, Melanie L. Bailey^2,3^, Marco A. Marra^2,3,5,6*^

^1^Genome Science and Technology Graduate Program, University of British Columbia, Vancouver, BC, Canada

^2^Michael Smith Laboratories, University of British Columbia, Vancouver, BC, Canada

^3^Canada’s Michael Smith Genome Sciences Centre, Provincial Health Services Authority, Vancouver, BC, Canada

^4^Department of Medical Oncology, BC Cancer, Vancouver, BC, Canada

^5^Department of Medical Genetics, University of British Columbia, Vancouver, BC, Canada

^6^BC Cancer Research Institute, BC Cancer, Vancouver, BC, Canada

*** Correspondence:**Corresponding Author
mmarra@bcgsc.ca

# Supplementary Figures and Tables

## Supplementary Tables

## Supplementary Table 1: Patient characteristics and treatments received prior to biopsy

| ID | Biopsy Site | Tumor Type | Histology | Chemo | Targeted | RT* |
| --- | --- | --- | --- | --- | --- | --- |
| POG650_1 | lung metastasis | Skin | Mucosal melanoma |  |  |  |
| POG217_1 | neck - skin metastasis | Gastrointestinal | Anal canal squamous cell carcinoma | Y |  | Y |
| POG217_2 | chest - skin metastasis | Gastrointestinal | Anal canal squamous cell carcinoma | Y |  | Y |
| POG130_1 | spinal metastasis | Colorectal | Adenocarcinoma, mucinous differentiation | Y |  |  |
| POG130_3 | spinal metastasis | Colorectal | Adenocarcinoma, mucinous differentiation | Y |  |  |
| POG130_4 | spinal metastasis | Colorectal | Adenocarcinoma, mucinous differentiation | Y |  |  |
| POG196 | pleural metastasis | Thymus | Thymic lymphoepithelioma-like carcinoma | Y | mTORi | Y |
| POG643_1 | brain recurrence | Central nervous system | Pleomorphic xanthoastrocytoma |  |  | Y |
| POG643_2 | brain recurrence | Central nervous system | Pleomorphic xanthoastrocytoma |  | BRAF/  MEKi |  |
| POG1128_2 | lung metastasis | Connective tissue | Synovial sarcoma | Y |  | Y |
| POG415_1 | intradural primary | Connective tissue | Extraosseous osteosarcoma |  |  | Y |
| POG415_2 | intradural recurrence | Connective tissue | Extraosseous osteosarcoma | Y |  |  |

## *RT: Radiation therapy

## Supplementary Table 2: Number and proportion of genes included in 3’ scRNA-seq and Flex broken down by chromosome

| **Chromosome** | **Number of genes in 3’ scRNA-seq reference** | **Number of genes probed by Flex** | **Proportion (Flex / 3’ scRNA-seq)** |
| --- | --- | --- | --- |
| chr1 | 3409 | 1934 | 0.567321795 |
| chr2 | 2540 | 1177 | 0.463385827 |
| chr3 | 1892 | 1010 | 0.533826638 |
| chr4 | 1533 | 714 | 0.465753425 |
| chr5 | 1810 | 833 | 0.460220994 |
| chr6 | 1827 | 941 | 0.515051998 |
| chr7 | 1686 | 852 | 0.505338078 |
| chr8 | 1495 | 626 | 0.418729097 |
| chr9 | 1318 | 732 | 0.55538695 |
| chr10 | 1393 | 696 | 0.499641062 |
| chr11 | 2065 | 1232 | 0.596610169 |
| chr12 | 1928 | 967 | 0.501556017 |
| chr13 | 788 | 301 | 0.381979695 |
| chr14 | 1474 | 587 | 0.398236092 |
| chr15 | 1266 | 546 | 0.431279621 |
| chr16 | 1649 | 770 | 0.466949666 |
| chr17 | 1992 | 1092 | 0.548192771 |
| chr18 | 781 | 262 | 0.33546735 |
| chr19 | 2027 | 1346 | 0.66403552 |
| chr20 | 964 | 511 | 0.530082988 |
| chr21 | 555 | 203 | 0.365765766 |
| chr22 | 900 | 419 | 0.465555556 |
| chrM | 13 | 12 | 0.923076923 |
| chrX | 1146 | 749 | 0.653577661 |
| chrY | 111 | 21 | 0.189189189 |

**Supplementary Table 3: Number of cells pre- and post-quality control for FFPE Flex and 3’ scRNA-seq**

|  | **Pre-filtering** | | **Post-filtering** | |
| --- | --- | --- | --- | --- |
| **Sample** | **FFPE Flex** | **3’ scRNA-seq** | **FFPE Flex** | **3’ scRNA-seq** |
| **POG1128_2** | 4478 | 7622 | 4418 | 7552 |
| **POG130_1** | 559 | 5640 | 554 | 5339 |
| **POG130_3** | 4836 | 3813 | 4763 | 3765 |
| **POG130_4** | 2109 | 7002 | 2061 | 6793 |
| **POG196** | 9392 | 6937 | 9243 | 6761 |
| **POG217_1** | 7174 | 4286 | 7085 | 4161 |
| **POG217_2** | 6678 | 5676 | 6572 | 5501 |
| **POG415_1** | 6031 | 4254 | 5967 | 4192 |
| **POG415_2** | 6335 | 4620 | 6246 | 4370 |
| **POG643_1** | 6672 | 1752 | 6540 | 1716 |
| **POG643_2** | 6850 | 6039 | 6745 | 5888 |
| **POG650_1** | 4232 | 4727 | 4184 | 4601 |

## Supplementary Figures


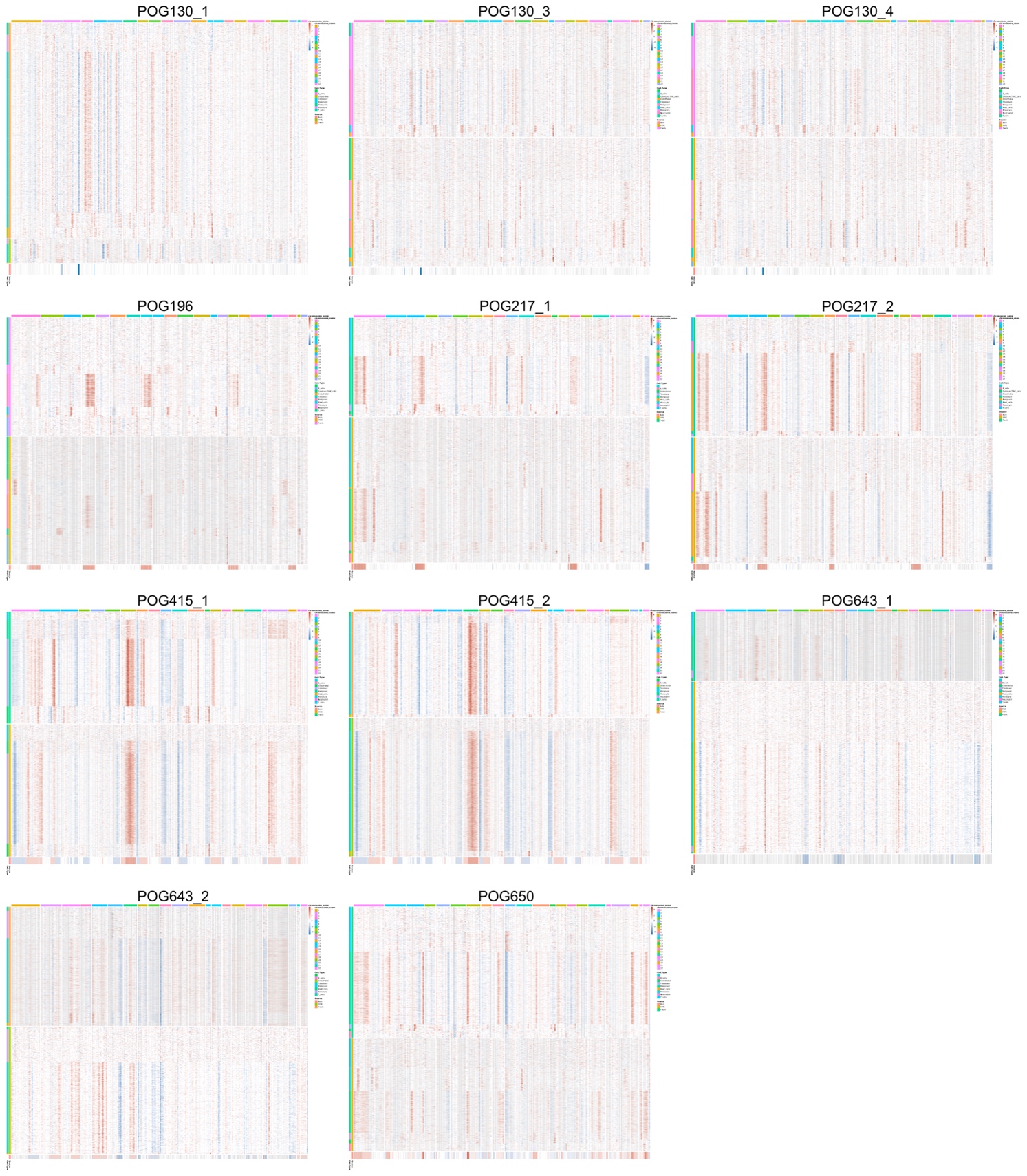


**Supplementary Figure 1: InferCNV results.** Heatmaps of predicted CNA values from 3’ scRNA-seq data, FFPE Flex data, and bulk WGS.


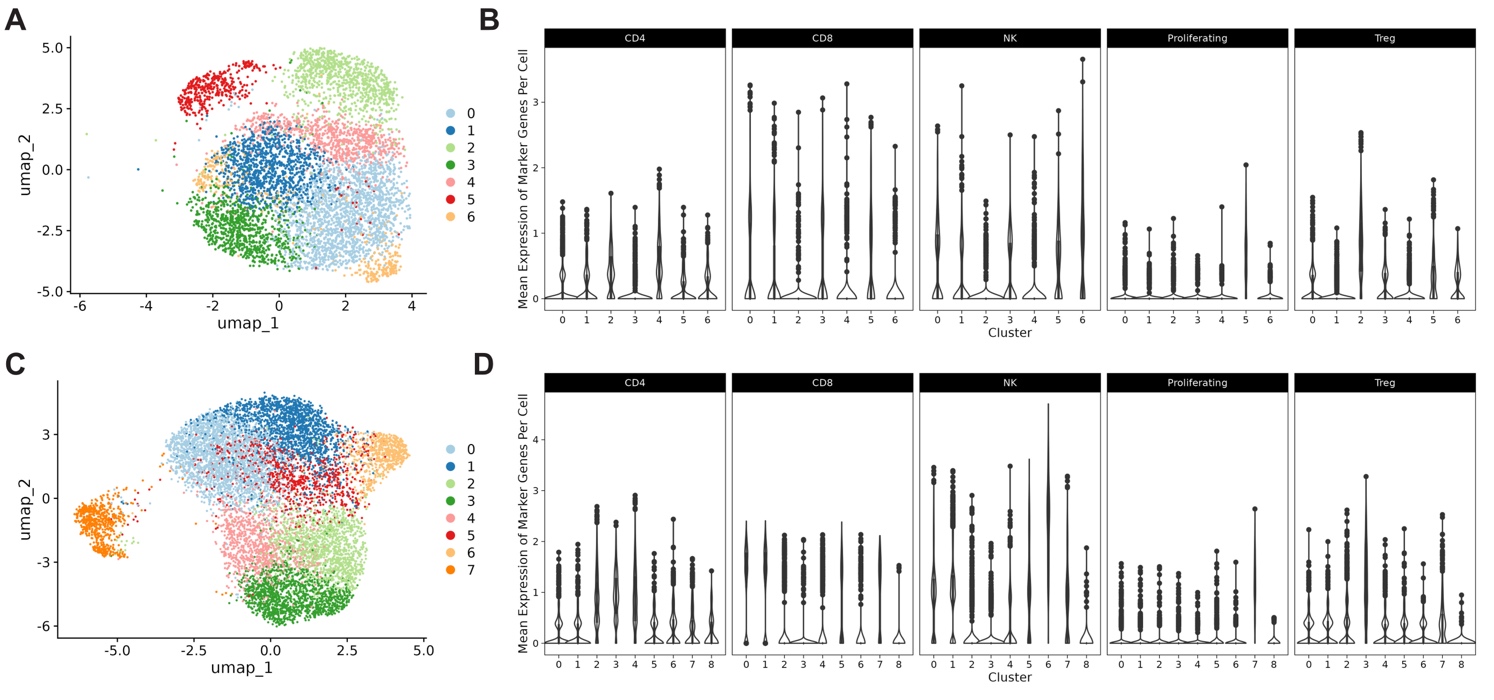


**Supplementary Figure 2: T cell subtyping.** (A, C) Batch corrected UMAPs of T cells from all (A) 3’ scRNA-seq and (C) FFPE Flex samples coloured by cluster. (B, D) Violin plots showing mean expression of marker genes for each T cell subtype across (B) 3’ scRNA-seq and (D) FFPE Flex clusters.
